# Neurocognitive function impairment after whole brain radiotherapy for brain metastases: actual assessment

**DOI:** 10.1186/1748-717X-7-77

**Published:** 2012-05-28

**Authors:** Agnes V Tallet, David Azria, Fabrice Barlesi, Jean-Philippe Spano, Antoine F Carpentier, Antony Gonçalves, Philippe Metellus

**Affiliations:** 1 Department of Radiation Oncology, Institut Paoli Calmettes, Marseille, France; 2 Department of Radiation Oncology and INSERM U896, CRLC Val d'Aurelle, Montpellier, France; 3 Multidisciplinary Oncology and Therapeutic Innovations Department & Centre Investigation Clinique, Aix Marseille University -Assistance Publique Hôpitaux de Marseille, Marseille, France; 4 Department of Medical Oncology, GH Pitié-Salpêtrière, Université Paris 6, Paris, France; 5 Department of Neurology, Hopital Avicenne, Assistance Publique des Hopitaux de Paris, Bobigny, France; 6 Department of Medical Oncology, Institut Paoli Calmettes, Centre de Recherche en Cancérologie de Marseille, UMR1068 INSERM, Aix-Marseille University, Marseille, France; 7 Department of Neurosurgery, Hopital La Timone, Assistance Publique-Hôpitaux de Marseille and INSERM UMR 911, Marseille, France; 8 Groupe de Réflexion sur la Prise en Charge des MétAstases Cérébrales (GRPCMaC, Marseille, France

**Keywords:** Neurocognitive impairment, Whole brain radiation therapy, Brain metastases

## Abstract

Whole brain radiation therapy (WBRT) is an effective treatment in brain metastases and, when combined with local treatments such as surgery and stereotactic radiosurgery, gives the best brain control. Nonetheless, WBRT is often omitted after local treatment due to its potential late neurocognitive effects. Publications on radiation-induced neurotoxicity have used different assessment methods, time to assessment, and definition of impairment, thus making it difficult to accurately assess the rate and magnitude of the neurocognitive decline that can be expected. In this context, and to help therapeutic decision making, we have conducted this literature review, with the aim of providing an average incidence, magnitude and time to occurrence of radio-induced neurocognitive decline. We reviewed all English language published articles on neurocognitive effects of WBRT for newly diagnosed brain metastases or with a preventive goal in adult patients, with any methodology (MMSE, battery of neurcognitive tests) with which baseline status was provided. We concluded that neurocognitive decline is predominant at 4 months, strongly dependant on brain metastases control, partially solved at later time, graded 1 on a SOMA-LENT scale (only 8% of grade 2 and more), insufficiently assessed in long-term survivors, thus justifying all efforts to reduce it through irradiation modulation.

## Introduction

Neurocognitive effect of whole brain radiation therapy (WBRT) is a crucial problem since fear has led physicians to postpone this effective treatment for patients with oligometastic brain metastases (BM) after local treatment (stereotactic radiosurgery (SRS) or surgery), despite its ability to reduce intracranial failure and neurologic death rates [1-3]. This seems detrimental, especially in patients for whom extra-cranial disease is absent or controlled and the primary tumor radiosensitive. Indeed, in situations of controlled extra-cranial disease, the objective of the treatment of newly diagnosed BM is not only to palliate symptoms, but also and mainly to control BM, and thus an aggressive approach is required. This is all the more important since, while randomized controlled trials (RCT) evaluating the omission of up-front WBRT did not show a difference in overall survival [1-3], some retrospective trials concerning patients without extra-cranial disease, have shown the opposite [4,5].

Determining the impact of WBRT on neurocognitive function (NCF) would provide support for therapeutic decision making for individual patient, for which we need to elucidate the incidence, time course, intensity, domains of neurocognitive changes following WBRT and their actual impact on patient quality of life (QOL). There is a paucity of data on neurocognitive impairment after WBRT, which has previously been assessed using various different neuropsychological tests, as well as different definitions of neurocognitive impairment. It is noteworthy that NCF is affected by a number of factors (i.e. BM volume, disease progression [intra and/or extra-cranial progression], chemotherapy, hormonotherapy, surgery, radiation, prior neurologic disease, medications, paraneoplastic effects, etc.) which should be considered when evaluating of the actual neurocognitive effect of treatments such as WBRT.

The objective of this review was to obtain accurate data regarding neurocognitive impairment after WBRT and to objectively include them in decision making at time of BM onset. We carried out a literature review of NCF tools used and their results.

Before the year 2000, most studies concerning the neurocognitive effects of WBRT were retrospective, thus without neurocognitive assessment by validated tests or without reliable baseline evaluation. These studies revealed potential dementia, and/or imaging changes [6-9].

Since 2003, neurocognitive tests have been included in RCT, however, the real impact of WBRT on NCF remains unclear because of the variability in time to assessment, definition of neurocognitive impairment, patient population, radiation doses and especially dose per fraction which is known to be a determinant in late side effects [6].

## Methods

### Search strategy

We carried out a literature review and analysed all English language published articles on neurocognitive effects of WBRT alone (without any concurrent treatment) for newly diagnosed BM or with preventive goal in adult patients, in either a prospective or retrospective setting, whatever the methodology used (Mini-Mental State Examination (MMSE) [10], battery of neurcognitive tests as described by Meyers and Brown [11]) provided there was a baseline assessment and results were compiled.

We conducted a systematic search using the Medline database up to September 2011. Eligible studies investigated WBRT in one of the study arm. The term “neurocognitive function” combined with the terms “irradiation” and “brain metastases” were used. Relevant articles were reviewed, and the reference lists from these sources were manually searched for additional relevant trials.

Articles were excluded from this literature review if they were individual case reports or review articles. Data extracted from studies included: number of patients accrued and evaluated in the WBRT arm of the study, radiation therapy schedule, disease status when available, neurocognitive tests used, definition of neurocognitive impairment, time of assessment, percentage of patients who experienced a neurocognitive decline. Data extracted from text, tables and figures of the articles were then tabulated.

## Results

We identified 16 articles reporting 12 trials assessing the neurocognitive outcome of patients treated with WBRT alone (6 based on the MMSE, 9 on a battery of validated neurocognitive tests, and one using the EORTC-Radiation Therapy Oncology Group [RTOG] Late Effects Normal Tissue [LENT]-Subjective, Objective, Management, analytic [SOMA] LS scale). Two of these articles assessed the neurocognitive impairment of WBRT following SRS. One of them used the MMSE as NCF assessment tool [12], the second was the only RCT to have neurocognitive outcome as primary end-point [13].

### Assessment on MMSE

The initial assessments of neurocognitive impairment after WBRT were based on MMSE measurement, both in therapeutic cranial irradiation (TCI) (4 studies), and in prophylactic cranial irradiation (PCI) (1 study).

#### *Neurocognitive outcome after TCI (BM present)*

The impact of fractionation schedule (accelerated hyperfractionation vs. standard accelerated fractionation WBRT) was analysed in a phase III study from the RTOG [14]. The primary end-point was overall survival. Murray et al., and Regine et al., evaluated the MMSE in 182 patients with BM enrolled in the accelerated fractionation arm (30 Gy/10 fractions) [15,16]. The authors found that 54.5% of patients experienced an improvement in their MMSE at any follow-up visit. At two and three months, 29% and 16% of patients respectively with pretreatment MMSE 28 to 30 experienced a decline. For patients with pretreatment MMSE <28, an improvement in MMSE was observed in 42% of patients at two months, 33% at three months, the MMSE was stable in 28% at two months, 18% at three months, and declined in 28% of patients at two months, and 46% at three months [16] (Table 1). Only the neurocognitive outcome of patients in the accelerated fractionation arm was extracted from these reports, but no difference was found between the two arms. It is noteworthy that a clinically/statistically meaningful drop in MMSE at two and three months was only observed in patients with uncontrolled BM. The authors pointed out the limitations of their secondary analysis due to the poor survival of the patient population, and thus the evaluation of neurocognitive effects of WBRT was restricted to the short term.

**Table 1:** Neurocognitive impairment after WBRT as assessed by MMSE

Study	N	Radiation scheme	Brain control	Assessment time				
				% of patients impaired				
				2month	3mo	6mo	12mo	18mo
Regine et al., 2001	182	30 Gy/10 fractions/12	Yes	0	0	NR	NR	NR
[16]		days (TCI)	No	28	46			
Shibamoto et al., 2008 [17]	92	40 Gy/20 fractions/33 days (TCI)	Yes	NR	7.4	11	12	0
Corn et al., 2008 [18]	92	37.5 Gy/15fractions/19 days (TCI)	Yes	18	24	24	28	28
			No	23	23	33	38	40
Aoyama et al., 2007 [12]	41	30 Gy/10 fractions/12 days (TCI)	NR	5 ^a^	16^a^	16 ^a^	28 ^a^	40 ^a^
				2 ^b^	10 ^b^	14 ^b^	21 ^b^	21 ^b^
				5 ^c^	14 ^c^	16 ^c^	24 ^c^	24 ^c^
Sun et al., 2011 [19]	340	30 Gy/15 fractions/19 days (PCI)	NR	NR	36	28	23	NR

Only studies from which percentages of patients impaired on NCF could be extracted are reported in this table. WBRT = Whole Brain Radiation Therapy; TCI = Therapeutic Cranial Irradiation; PCI = Prophylactic Cranial Irradiation; NR = not reported; ^a^ = patients who underwent a 3-point decrease in MMSE; ^b^ = patients who underwent a decrease of MMSE ≤ 26; ^c^ = patients who underwent a 3-point decrease in MMSE, excluding those who return to their initial MMSE.

The incidence of brain atrophy and decline in MMSE after WBRT [40 Gy in 20 fractions with (72% of patients) or without a 10 Gy boost] was prospectively described in 92 patients with BM [17]. A decrease in MMSE scores of ≥ 4 points was observed in 7.4%, 11%, 20%, 12%, 5.9% of assessable patients at 3, 6, 9, 12, and 15 months respectively, and no decrease in MMSE was observed thereafter (last assessment: 36 months) (Table 1). This was not correlated with brain atrophy. Patients with brain progression were excluded from the evaluation but patients with systemic progressive disease were included, and about half the patients with an MMSE score decrease had systemic progression and a decrease in performance status.

The efficacy of Thalidomide (a putative antiangiogenic agent) concurrently administered with WBRT was assessed in a phase III trial for 156 adult patients with multiple BM [18]. In 92 patients of the WBRT alone arm, the neurocognitive effect of WBRT (37.5 Gy in fractions of 2.5 Gy) was investigated. As shown in Table 1, 28% of patients with brain disease control experienced a neurocognitive decline at one year (but the magnitude of this decline was not given), while 38% of patients with intra-cranial progression showed a drop in MMSE scores from baseline.

Efficacy of WBRT added to SRS at initial treatment of newly diagnosed BM was investigated in a RCT from Aoyama et al.[1] The authors subsequently reported on the NCF deterioration rate of 36 patients (with initial MMSE ≥ 27) enrolled in the WBRT plus SRS arm [12]. The authors assessed the MMSE deterioration every 3 months, and recorded the percentage of patients who experienced a drop in MMSE score at least once, unless they recovered subsequently their initial MMSE, the percentage of patients impaired at a specified time, and the percentage of patients who experienced a decrease of MMSE ≤ 26 on at least one occasion. The results are presented in Table 1. Interestingly, no statistically significant difference was observed between patients treated with WBRT and SRS and those treated with SRS alone, despite a difference in the time to deterioration, which was longer for the combined treatment group (> 12 months for the WBRT + SRS group vs < 8 months for the SRS group, *p* = 0.05)). Finally, while a quarter of patients experienced a drop in MMSE at 12 months, this percentage worsened over time, indicating that WBRT is effective at preventing the neurocognitive decline resulting from brain tumor recurrence in the first 2 years, but could be a cause of continuous deterioration of NCF in long-term survivors.

#### *Neurocognitive outcome after PCI (no BM)*

The effect of prophylactic cranial irradiation (PCI) on NCF and QOL was assessed by Sun et al. [19] in 64 patients with small cell lung cancer (SCLC) without BM and without disease progression after completing definitive therapy. NCF was assessed with MMSE, and Hopkins Verbal Learning Test (HVLT), using the Reliable Change Index (RCI) method [20]. One third of patients experienced a decline in MMSE score 3 months after completion of PCI, while 28% and 23% were impaired on the same test at 6 and 12 months respectively (Table 1). Moreover, no significant difference in MMSE (except at 3 months) was observed at 1 year when comparing patients submitted to PCI and those without PCI. Conversely, the HVLT was more often impaired at 1 year in the PCI arm, with an average of 30% of patients impaired at 1 year (compared to 6% of patients in the observation arm), which is less than the impairment observed at 3 months (Table 2). The authors were unable to separate those patients who did or did not develop BM, due to the small number of events.

**Table 2:** Time, NCF domain impaired, and incidence of NC impairment after WBRT

Study	Radiation scheme	Nb	Assessment time					Domains impaired	Impairment definition
				% of patients impaired					
			1 mo	2 mo	3 mo	5 mo	12 mo				
Sun et al., 2011 [19]	PCI 30Gy/15 fr	163						^§^HVLT	
			_	_	45%		26%	Recall	
					44%		32%	Delayed recall	
Meyers et al., 2004 [22]	30 Gy/ 10fr ± MGd	401	_	_	31%	_		^*^Pegboard	>2 DS
					7%			^†^COWA	>2 DS
	30Gy/10fr alone	208					48%	^§^HVLT	≥ 4.5 DS
							48%	^†^COWA	≥ 4.5 DS
Chang et al., 2009 [13]	SRS + WBRT (30 Gy/12 fractions)	28			64%	28%		^§^HVLT	Drop ≥ 5 points
					22%			Delayed recall	
					11%			Delayed recognition	
Welzel et al., 2008 [27]	TCI: 40Gy/20fr	16	9%		_	_	_	^§^MCG	RCI (≥1score)
			18%	43%				Overall cognitive decline	RCI (≥2/12 test scores)
				57%	_	_	_	^§^AVLT	RCI (≥1score)
									
	PCI: 36Gy/18fr	13	23%		_	_	_	^§^MCG	RCI (≥1score)
			8%	11%				Overall cognitive decline	RCI (≥2/12 test scores)
				44%				^§^AVLT	RCI (≥1score)
					_				
Van Oosterhout et al., 1995 [28]	PCI (30 Gy / 15 fractions)	5	0		_	0	_	none	> ?DS
Komaki et al., 1995 [29]	PCI (25 Gy / 10 fractions)	11	_	_	_	_	0	none	≥ 1.5 DS
Wolfson et al., 2010 [31]	PCI								
	25 Gy/10 fr	131					62%	^§^HVLT, ^†^COWA, ^‡^TMT-A and B	Decrease in at least one of the test (from baseline) (RCI)
	36 Gy/18 fr	67					85%		
	36 Gy/twice-daily 24fr	66					89%		

Only studies from which percentages of patients impaired on NCF could be extracted are reported in this table. PCI = Prophylactic Cranial Irradiation; MGd = Motexafin Gadolinium; ^*^Pegboard: grooved pegboard test, examining motor speed and dexterity; ^†^COWA: Controlled Oral Word Association, test examining verbal fluency; ^§^ HVLT: Hopkins Verbal Learning Test, examining immediate recall, delayed recall, and recognition; MCG: Medical College of Georgia Complex Figures, examining visual memory and visual construction (copy, immediate recall and delayed recall); AVLT: Auditory Verbal Learning Test, examining verbal memory; ^‡^TMT-A and B: trailmaking test A, examining visual-motor scanning speed, and B, examining executive functions.

### Assessment on a battery of validated neurocognitive tests

A report by Meyers and Brown, to identify the optimal battery of neurocognitive tests to use for patients with central nervous system disease, did not recommended the use of brief mental status evaluation, which only detect significant dementia and have extremely poor sensitivity in detecting neurocognitive impairment in brain tumor patients [11].

#### *Neurocognitive outcome after TCI (BM present)*

The effect of Motexafin Gadolinium (MGd) combined with WBRT (30 Gy given in 10 daily fractions) was evaluated in 401 patients with BM in a multi-institutional phase III study [21-23]. The co-primary end-points were survival and time to neurologic progression. As a secondary end-point, the authors evaluated the time to neurocognitive progression. The WBRT alone arm included 208 patients, and their neurocognitive outcome was analysed (Table 2). Findings from these reports were: (1) nearly all patients (90.5%) with multiple metastases (80.1%) had some evidence of neurocognitive impairment at baseline (impairment of one or more neurocognitive tests), 42,4% having an impairment in four or more tests; (2) this baseline impairment of neurocognitive function was highly correlated to the volume of the indicator lesion at baseline; (3) patients progressed most in pegboard (fine motor) performance at three months; (4) at four months, there was a sharp drop in the mean NCF scores, mainly in memory function, while at 15 months, the mean NCF test scores gradually improved, especially in verbal fluency, executive function and fine motor coordination, but not memory; (5) at 12 months, 48% of surviving patients treated by WBRT alone (with or without progressive disease) had impaired HVLT and Controlled Oral Word Association Test (COWAT); (6) median time to NCF deterioration for all eight tests was longer in good responders than in poor responders (p = 0.008), especially for executive function and fine motor coordination.

Chang et al. described the only RCT with NCF as primary end-point [13]. Patients with 1–3 BM were randomly assigned to SRS alone (30 patients) or SRS plus WBRT (28 patients). The RCI was used to measure meaningful change between baseline and four months for the different tests used; a decline was declared if there was a decline in the total recall score of ≥5 points compared with baseline. The trial was halted by the data monitoring committee according to the early rules on the basis that total recall at 4 months for SRS + WBRT group was inferior to SRS alone group. Of the patients receiving combined treatment, 64% experienced a decline in total recall tests. Extensive discussions of these results highlighted several shortcomings [24,25], and no definite conclusions could be drawn. In particular, patients in the combined treatment group had a surprisingly, unexplained short median survival which could have had an impact on NCF tests and time of assessment is debatable, since a transient effect on memory following WBRT has already been proved, with a nadir at 4 months [26].

Welzel et al. published a prospective evaluation of neurocognitive effect of WBRT for 44 patients with or without BM, irradiated prophylactically (PCI: 13 patients) or for therapeutic effect (TCI: 16 patients), compared with a control group irradiated on the breast (15 patients) [27]. Patients were described as improved, stable or impaired on NCF using the RCI method. NCF was assessed at baseline, at the “acute phase” (after starting radiotherapy and at its completion), and at the “subacute phase” (at 6–8 weeks after radiotherapy) (Table 2). Approximately one third of the TCI and PCI patients had cognitive impairment on at least two subsets even before treatment. At the end of radiotherapy, almost all verbal memory scores had improved or returned to the baseline level. Declines in visual memory were balanced by improvements. The authors concluded that cognitive dysfunction after WBRT restricted to verbal memory.

#### *Neurocognitive outcome after PCI (no BM)*

The studies from Van Oosterhout et al. [28], Komaki et al. [29], Grosshans et al. [30], evaluating the neurocognitive outcome of patients subjected to PCI included few patients and the results lacked of precision. Thus, the data from these studies were not detailed, but it was noteworthy that the authors did not observe any consistent neurocognitive impairment after PCI (Table 2).

Impact of different total dose and schedule of PCI on neurologic deterioration was analysed by Wolfson et al. for 264 patients with limited-disease SCLC [31]. Patients who achieved a complete response after chemotherapy and thoracic irradiation, without evidence of brain disease on imaging were randomized to one of the three arms of PCI (25 Gy/10 fractions or 36 Gy/18 fractions or 36 Gy/twice-daily 24fractions). The NCF assessment (co-primary end-point) was performed at baseline, 6 months and 12 months after randomization. Neurologic deterioration was defined as a decrease of one SEM from baseline in any of the battery of tests, and confirmed using the RCI. More than 60% of patients receiving 25 Gy and 80-90% of those having 36 Gy PCI had documented neurotoxicity at one year (as defined above) (Table 2). The second co-primary end-point was QOL measured by the EORTC QLQ-C30 and brain module (BN20). There was no impact of this neurologic deterioration on QOL measurements at 12 months. Moreover, the neurocognitive outcome of the patients included in this series was assessed using the LS scale [32]. LS memory decline at 3 years was more frequent with grade 1 deficit (decreased short term, difficulty with learning), as was observed in 44% of patients, than grade ≥2 deficit observed in 8% of patients without intra-cranial progressive disease. The authors concluded that mild deterioration of some items, such as memory, intellectual deficit and cognitive functions possibly related to PCI, should be balanced by considering the beneficial effects of PCI on survival and incidence of BM.

## Discussion

Radio-induced neurocognitive impairment evolves in a biphasic pattern: a subacute transient decline with a peak at four months, and a late delayed irreversible impairment of NCF several months or years after completion of WBRT [26,33-35]. The objective of this review was therefore to identify the incidence of these disorders in the two phases in order to provide support for therapeutic decision making. The first phase is of importance for patients with worse prognoses (due to progression of extra-cranial disease), whereas for long-term survivors, the late delayed impairment will strongly influence the therapeutic decision.

Of course, the NCF decline is not exclusively due to WBRT, and as mentioned above, several factors may make it worse. Moreover, pre-treatment cognitive status (highly correlated to the volume of the disease) has significant effects on neurocognitive outcome, as reported by Meyers et al. [22].

*Neurocognitive decline in patients without visualizable brain disease*. This literature review shows that for patients with brain disease control, the risk of neurocognitive impairment as assessed by MMSE is low (0 to 24% of patients impaired at 3 months, 12 to 28% at one year). A lower incidence of a drop in MMSE was observed by Regine et al. [16], and Shibamoto et al. [17] compared to that observed by Corn et al. [18], which may simply reflect the difference in defining the decline in MMSE, which was a decrease in MMSE scores of ≥ 4 points and a decrease of ≥ 3 points respectively for the two first publications, and only one point or more for the last. Most studies assessing NCF in the setting of PCI (with lower total doses and dose per fraction than therapeutic cranial irradiation) showed very low incidence of neurocognitive impairment at one year. This had previously been observed in a study investigating the benefit of PCI for non small cell lung cancer, in which neurocognitive decline was assessed using a battery of neurocognitive tests (without baseline evaluation) [36]. The authors found not significant difference for long term survivors in patients with or without PCI. Similar results were reported by Arriagada et al. in a trial assessing the efficiency of PCI for SCLC [37].

*Neurocognitive decline in patients with BM present.* Based on Table 1, NCF as assessed by MMSE score is impaired after WBRT in the year following the completion of WBRT, but appears strongly related to uncontrolled brain metastases. Later assessments were not available, except in the study from Aoyama et al., which appeared to have shown that neurocognitive decline occurred continuously over time. For this reason, the authors subsequently attempted to isolate prognostic factors for brain recurrence in patients with expected long survival, with the objective to withhold WBRT [38,39]. They found that patients who had solitary BM in the absence of extracranial metastases were at lower risk of brain tumor recurrence compared with patients with multiple BMs or extracranial disease, and proposed to postpone WBRT in those cases, given that the brain tumor recurrence risk was 31% at 6 and 12 months in patients at lower risk without “up-front” WBRT, allowing 69% of patients to avoid unnecessary treatment.

When patients were assessed through a battery of neurocognitive tests, almost half of patients were affected by a decline, namely 31 to 57% at 3 months, and 48 to 85% at one year, depending on the definition of impairment (Table 2).

WBRT-induced neurocognitive toxicity is to be balanced against the neurocognitive toxicity that may cause brain disease recurrence. Some publications describe a correlation between brain failure and neurocognitive decline and subsequently altered QOL [12,21-23,40,41]. These prospective clinical trials demonstrate a potential benefit of successful WBRT (i.e., achieving intracranial disease control) on NCF-preservation [16,22]. The authors highlighted the fact that patients who demonstrated good radiologic response to WBRT had improved executive function and fine motor coordination, but not memory, suggesting that WBRT might specifically impair hippocampus-related functions, leading to the concept of hippocampus avoidance during WBRT. One RCT (from Virginia Commonwealth University) with this objective was undertaken but unfortunately was closed for lack of accrual (only 8 patients enrolled). Two phase II trial (RTOG: http://clinicaltrials.gov/ct2/show/ NCT01227954, NCT01414738) and one phase III trial (France: http://www.sante.gouv.fr/IMG/pdf/resultats_PHRC_2011_cancer-2.pdf) are ongoing.

## Conclusion

Neurocognitive impairment is of concern in the setting of WBRT delivered for BM. WBRT after local treatment (surgery or SRS) allows the best brain disease control to be obtained to date. Thus, it is necessary to minimize its late side effects, and thereby patient enrollment in clinical trials for WBRT needs to be expanded so that multiple methods for avoiding and mitigating acute and late toxicities may be developed. Local treatment alone for oligometastatic brain disease is an option since postponing WBRT has not shown significant impact on overall survival. The results of this review will enable physicians to inform patients about benefits and risks of these two treatment options.

## Abbreviations

WBRT: Whole brain radiation therapy; BM: Brain metastases; SRS: Stereotactic radiosurgery; RCT: Randomized controlled trials; NCF: Neurocognitive function; QOL: Quality of life; MMSE: Mini-Mental State Examination; EORTC: European Organisation for Research and Treatment of Cancer; RTOG: Radiation Therapy Oncology Group; LENT: Late Effects Normal Tissue; SOMA: Subjective, Objective, Management, analytic; LS scale: SOMA-LENT scale; PCI: Prophylactic cranial irradiation; SCLC: Small cell lung cancer; HVLT: Hopkins Verbal Learning Test; RCI: Reliable change index; MGd: Motexafin Gadolinium; COWAT: Controlled Oral Word Association Test; TCI: Therapeutic cranial irradiation; SEM: Standard error of mean; EORTC QLQ-C30: EORTC quality of life questionnaire.

## Competing interest

AT received honoraria from Roche, DA received honoraria from Roche, Sanofi and Novartis, FB received honoraria from Roche, AC is consultant for Roche, AG received honoraria and funding supports from Roche, GSK and Astra Zeneca, PM received honoraria from Roche, JPS received honoraria or funding supports from: Roche, GSK, Merck, and Astra Zeneca, We are not actually sure that this could constitute any conflict of interest in connection with this manuscript.

## Authors’ contributions

AT and PM performed the literature search and wrote the manuscript. JPS, AFC and AG performed critical revision. AD and FB participated in the conception as well as the preparation of the manuscript; All authors read and approved the final manuscript.
